# Intrinsic Dependence of Groundwater Cation Hydraulic and Concentration Features on Negatively Charged Thin Composite Nanofiltration Membrane Rejection and Permeation Behavior

**DOI:** 10.3390/membranes12010079

**Published:** 2022-01-10

**Authors:** Miroslav Kukučka, Nikoleta Kukučka Stojanović

**Affiliations:** EnviroTech d.o.o., Sterije Popovića 42, 23300 Kikinda, Serbia; nikol@envirotech.rs

**Keywords:** cations permeation mechanism, steric hindrance factors, membrane rejection affinity, Gibbs free energy

## Abstract

Commercial nanofiltration membranes of different molecular weight cut-offs were tested on a pilot plant for the exploration of permeation nature of Ca, Mg, Mn, Fe, Na and ammonium ions. Correlation of transmembrane pressure and rejection quotient versus volumetric flux efficiency on nanofiltration membrane rejection and permeability behavior toward hydrated divalent and monovalent ions separation from the natural groundwater was observed. Membrane ion rejection affinity (MIRA) dimension was established as normalized TMP with regard to permeate solute moiety representing pressure value necessary for solute rejection change of 1%. Ion rejection coefficient (IRC) was introduced to evaluate the membrane rejection capability, and to indicate the prevailed nanofiltration partitioning mechanism near the membrane surface. Positive values of the IRC indicated satisfactory rejection efficiency of the membrane process and its negative values ensigned very low rejection affinity and high permeability of the membranes for the individual solutes. The TMP quotient and the efficiency of rejection for individual cations showed upward and downward trends along with flux utilization increase. Nanofiltration process was observed as an equilibrium. The higher the Gibbs free energy was, cation rejection was more exothermic and valuably enlarged. Low Gibbs free energy values circumferentially closer to endothermic zone indicated expressed ions permeation.

## 1. Introduction

Dominant constituents of groundwater include inorganic salts represented by hydrated cations and different anions as counter ions. Most commonly found are cations of calcium, magnesium, iron, manganese, sodium, and ammonium [1]. Representing anions are bicarbonate, chloride, sulfate, and phosphate. Besides those mentioned, many other chemical elements as ions and dissolved organic matter are present in natural underground water solution, known as groundwater. The chemical composition of groundwater is often a consequence of the water source environment, i.e., soil and strata geological nature [2].

Pressure-driven process with nanofiltration (NF) membranes is one of the most utilized ways for successful groundwater softening and partial demineralization [3], as well as drinking water production [4]. Reverse osmosis is also a commonly used pressure-driven process for groundwater treatment. Comparison of nanofiltration and the reverse osmosis process was conducted recently by Cai et al., where Sr^2+^ and natural organic matter rich water was treated with applied pressure and pH. It was reported that overall better rejection was obtained using reverse osmosis membranes, probably due to the nanofiltration membranes’ large pore size [5]. On the other hand, nanofiltration is proven to be suitable when specific permeate composition is needed. As reported by Riuz-García et al., if requirements for total dissolved solids or some specific harmful ions in the permeate are not as high, in the case of irrigation, the use of nanofiltration alone or in combination with brackish water reverse osmosis could reduce operating costs in terms of energy consumption [6]. 

The basic principle of nanofiltration salt concentration in water solution is defined by membrane rejection characteristics [7]. Salt rejection also significantly depends on the true membrane charge. Researchers in a recently reported study fabricated a positively charged nanofiltration membrane that was used for effective separation of Mg^2+^ and Li^+^ from salt-lake brine with a high Mg^2+^/Li^+^ mass ratio [8]. Jin et al. developed a positively charged nanofiltration membrane of excellent stability via interfacial polymerization reaction on a polyethersulfone substrate used for water softening [9]. Crossflow filtration rejects inorganic hydrated cations depending on electrical charge, valence, and hydration degree, as well as their concentration and possible interactions with other present ions. These ions hydrated by noncovalent interactions exhibit behavior analogous to gel structures and can change their shapes near membrane surface under the influence of transmembrane pressure [10]. This phenomenon is a consequence of loss or rearrangement of water molecules in the hydration shells [11]. Temperature also has a large effect on the transport through the membrane. As presented by Roy and Lienhard, the membrane structural changes and changes in membrane charge with increase in temperature cause increase and decrease of the permeate concentration, respectively [12].

Scaling presents a large issue for membrane pressure-driven processes. Namely, the scaling potential is directly influenced by concentration of soluble salts in the feedwater, temperature, and pH value, as well as by the membrane system recovery rate. There are many papers regarding scaling problems [13,14]. One of the ways to reduce scaling is usage of anti-scaling solutions that provide very good results [15]. Novel methods are being developed for better prediction and scaling problem solving. One such method is an algorithm that takes into account the scaling potential of SiO_2_, CaCO_3_, CaSO_4_, BaSO_4_, SrSO_4_, and CaF_2_ using different kind of anti-scalant products and was developed for calculation of maximum water recoveries of different groundwater bodies in the Gran Canarias and Tenerife Islands. The algorithm also allows the user to fix the solubility limits below those established by the different manufacturers [16]. Another method for prediction of the operational limits of nanofiltration modules working in high scaling risk situations was developed by Mitko et al. [17]. The authors of this paper did not include any scaling measurements, tracking, evaluating, or calculating in their specific research.

Polymeric asymmetric thin-film composite nanofiltration membranes are slightly negatively charged. The base of NF membranes is the surface of an ultrafiltration membrane which supports interfacial polymerization between a diamine and an acyl chloride to product-polyamide barrier layer [18]. Lately, novel sulfonated poly(aryl ether sulfone) composite nanofiltration membranes have been prepared in order to obtain better chlorine resistance and thermal stability [19]. The majority of available membrane types consist of ionizable amine and carboxylic functional groups which enable negative surface charge at a pH near neutral [20]. The consequence of fixed charged group existence is the repelling of ions with the same charge (co-ions) and, at the same time, attraction of counter ions carriers of opposite charge [21].

Previous research showed that better rejection of divalent ions is evident due to their larger hydrated size in comparison to monovalent cations [22], as well as rejection mechanisms [23,24]. Retention mechanisms in NF are mainly established based on size (steric properties) and Donnan (charge) exclusion [21,25]. Steric retention was cognized in cases of species with significantly larger hydrated sizes than the membrane pore size [26], while transport within the pores of species with sizes similar to that of the membrane pores may be hindered [27]. 

The fact that divalent ions are better removed than monovalent ones is well known and explored in the scientific community. For example, it was reported by Fang et al. that steric hindrance may dominate during the nanofiltration process of divalent Mg, resulting in high rejection of MgSO_4_ [28]. On the other hand, Zhao and Wang observed that a weaker electrostatic repulsive interaction to the positively charged membrane is found for Na^+^ in comparison with multivalent membranes [29]. However, there are not many papers dealing with nanofiltration permeation behavior using natural groundwater. The authors of this paper present a different approach to better understanding the permeation characteristics of the commercial polymeric asymmetric thin-film composite nanofiltration membranes when exposed to natural groundwater. 

The transport of solutes through an NF membrane in general should be explained as diffusion occurring in a complete pressure range but dominant at lower pressures, convection which is proportional to applied pressure [30], and electromigration [31]. Besides that, under low transmembrane pressure size exclusion is a dominant separation process [11]. The electric charge expressed through the electric field gradient is inherently part of the charged solutes nanofiltration transportation [32]. 

It can be found in the literature that all membrane processes are nonequilibrium processes [33]. The essential assumption of the partitioning mechanism, both near and at the membrane porous layer, is the availability of cations adsorption on the negatively charged membrane surface. On the whole, some ions are associated at the membrane surface, while some of them are distributed at the diffuse layer through to the bulk zone [34]. Assuredly, hydrated solutes are positioned within the interfacial area known as the Gibbs dividing surface [35] which is in the function of water molecules and size of all ions that passes through the membrane. Water molecule path length is longer than the trajectory for hydrated cations and is as shorter as the hydration ion sphere is relatively larger, as proposed by Shen et al. [36]. There are two possible coherent reversible processes of hydrated cation transport. These sustained phenomena are a dynamic electrostatic equilibrium between diffuse and interface layers, and among the interface layer and pore opening. Furthermore, individual cation hydrated radius and hydration energy determine the partitioning mechanism, as some cations are transported through the membrane channels and some are retained. Regarding this issue, investigations presented in this paper lean on the thought that the nanofiltration process can be an equilibrium one, i.e., that the processes occurring at the surface of the membrane, as well as in the membrane pores, can be observed as the steady environment. Additionally, the aim of this work was investigation of transmembrane pressure and rejection quotient correlation versus volumetric flux efficiency and their influence on nanofiltration membrane rejection and permeability behavior toward hydrated divalent calcium, magnesium, iron, and manganese, as well as monovalent sodium and ammonium ions originating from natural groundwater. These investigations were conducted by testing commercial NF membranes of different molecular weight cut-off (MWCO) and their combination in the pilot plant scale. The authors introduced the membrane ion rejection affinity (MIRA) dimension and ion rejection coefficient (IRC) to better understand and present the membrane rejection capability, and to indicate the nature of the ion separation mechanism at the membrane surface. 

## 2. Materials and Methods

### 2.1. Water Source

Nanofiltration experimental series were conducted in the City of Kikinda (45°49′46.99″ N, 20°27′55.01″ E), Serbia. For this purpose, water from a drilled well named “Sterija” from a second hydrological layer at the depth of 52 m was used. Selected physical–chemical characteristics of investigated groundwater are presented in Table 1. 

### 2.2. Nanofiltration Pilot Plant

**Figure 1:**
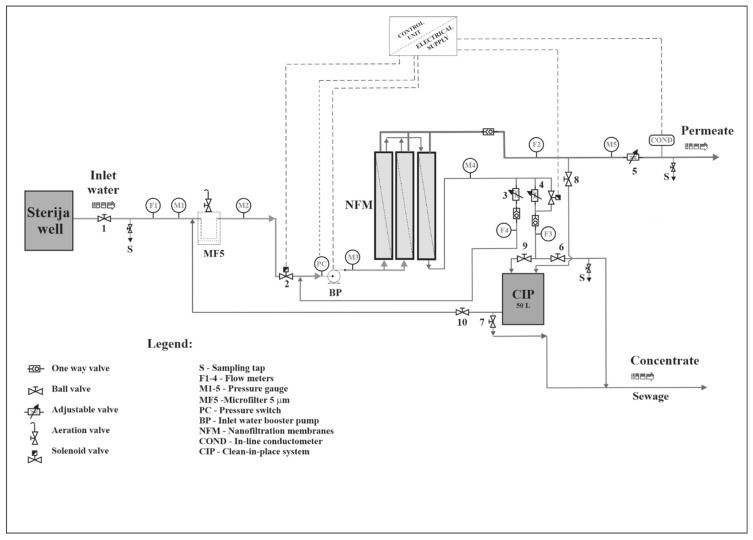
Schematic of the pilot plant.

A nanofiltration pilot plant (PNF) was designed in the research and development department of Envirotech, Kikinda, Serbia. PNF was manufactured using components presented in the Table 2. Maximum operating permeate flow was 1000 L/h. A detailed schematic of the pilot plant is given in the Figure 1. The pilot plant was fully automated, and it was operated by a programmable logic controller (PLC) in the following manner. After the power switch was turned on, the PLC opened the solenoid valve and the initial flushing of the membranes for the duration of 2 min began. After that, the booster pump was turned on, starting the filtration process. 

Experiments were conducted using well water that was distributed to the booster pump under the submersible pump pressure of up to 3 bar. After that, the booster pump reduced the pressure on the membranes. When the first experimental point was set up, i.e., when the first values for permeate, concentrate, and recirculate flows were fixed, the pilot plant was in operation for 30 min before the first experimental sample was taken. Every next experimental point and sample was obtained in the same manner. Adjustment of concentrate and recirculate flow rates at each experimental point contributed to the different feed water pressure and permeate flow rate. Additionally, it affected feed water and permeate fluxes, as well as membrane rejection of investigated solutes, which will be shown later in this work. 

Spiral-wound, thin-film composite anisotropic polyamide nanofiltration membranes were used. Membranes were produced by Toray Chemical Korea Inc., Seoul 07320, Korea. CSM-NE 4040-70 (NE70) and CSM-NE 4040-90 (NE90) types of membranes were used with defined MWCO [37] in order to evaluate the permeation mechanism of different ions with regard to the wide range of MWCO. As stated in the manufacturer’s specification, membranes NE70 and NE90 remove about 70% and 90% of total dissolved solids from inlet water, respectively. The effective area (A_m_) of each membrane type was 7.9 m^2^. These membranes have a relatively dense, extremely thin surface permselective layer that is supported on a more open, thick, porous structure. PNF was designed to allow a two-stage nanofiltration process. The experimental pilot device consisted of three nanofiltration elements, two of which were in the first stage. The second stage consisted of one membrane, and it was used for the filtration process of the concentrate obtained from the first stage. 

Three membrane combinations were selected for the investigation purposes, which represent different experimental series (Table 3). Effective pore radii (r_E_) in nm of the membrane systems were calculated at the base of the empirical correlation between r_E_ in nm, and membrane combination molecular weight cut-off (MWCO) in Da [30]:

(1) $$r_{E}=0.0325\cdot {\mathrm{MWCO}}^{0.438}$$

Obtained data for r_E_ were accepted as a single-pore radii, neglecting the pore size distribution.

Feed water pressures (P1) in bar, flow rates (Q_f_) in Lh^−1^, concentrate pressures (P2) in bar, permeate backpressures (P_p_) in bar, permeate flow rates (Q_p_) in Lh^−1^, recirculate flow rates (Q_r_) in Lh^−1^ as well as concentrate flow rates (Q_c_) in Lh^−1^ were monitored during the experimental procedure using flow meters and pressure gauges.

Permeate electrical conductivity (E_c_) was monitored on the in-line conductivity meter. Concentrations of Ca^2+^_(aq)_, Mg^2+^_(aq)_, Fe^2+^_(aq)_, Mn^2+^_(aq)_, Na^+^_(aq)_, and NH_4_^+^_(aq)_ ions in inlet water (C_i_) in mg L^−1^, permeates (C_p_) in mg L^−1^, and concentrates (C_c_) in mg L^−1^ were determined. Standard methods were applied using an atomic adsorption spectrophotometer from Shimadzu, Kyoto 604-8511, Japan, type AA-7000 with GFA, in order to determine Ca^2+^_(aq)_, Mg^2+^_(aq)_, Fe^2+^_(aq)_, Mn^2+^_(aq)_, and Na^+^_(aq)_ concentration. NH_4_^+^_(aq)_ ion was determined by ionic chromatograph DIONEX, Sunnyvale, CA, USA, type IC/ICS 3000. 

Every series reached up to six experimental points. The presented hydraulic parameters and physical–chemical analysis results are average values of three repetitions for each experimental point within each membrane combination. 

The clean-in-place (CIP) procedure was undertaken after the end of every series to ensure clean membrane surface at the beginning of the next experimental series. CIP was done by utilizing 2% citric acid and 0.2% NaOH, both over the duration of two hours. After the CIP was finished, the membranes were flushed with inlet water to ensure the membranes were free of cleaning solution, which was determined by pH monitoring.

### 2.3. Applied Calculations

Transmembrane pressure (TMP) originates as a pressure subtraction between the concentrate and permeate membrane sides. TMP in bar was calculated as presented in the Equation (2):

(2) $$\mathrm{TMP}=\frac{P1+P2}{2}-P_{P}$$

Feed (J_f_) and permeate (J_p_) fluxes, both in L·m^−2^·h^−1^, were calculated as shown in Equations (3) and (4):

(3) $$J_{f}=\frac{Q_{f}}{A_{m}}$$

(4) $$J_{p}=\frac{Q_{p}}{A_{m}}$$

Flux efficiency (FE), a dimensionless unit, was introduced as a measure of permeate flux utilization with regard to feed flux (Equation (5)).

(5) $$\mathrm{FE}=\left(1-\frac{J_{p}}{J_{f}}\right)$$

A solute rejection unitless parameter (R) is defined as the ratio of the amount of solute that passes through the membrane divided by the initial feed concentration (C_f_) in mg L^−1^ [38] (Equation (6)).

(6) $$R=\frac{c_{f}-c_{p}}{c_{f}}$$

Values of all investigated feed ion concentrations (C_f_) in mg L^−1^ were calculated as shown in Equation (7).

(7) $$c_{f}=c_{i}\times \frac{Q_{i}}{Q_{f}}+c_{c}\times \frac{Q_{r}}{Q_{f}}$$

Relative ionic permeability (RP), in %, represents the capability of the confined membrane channels to enable passing of solvent ions through the fine pores to the permeate side, and was calculated as [10]:

(8) $$\mathrm{RP}=\left(\frac{C_{p}}{C_{f}}\right)\times 100$$

The electrochemical equilibrium of feed and permeate cation molar concentrations occurred at each investigated sampling point. Separation process thermodynamic analysis was conducted at a constant temperature of 289 K and constant membrane pressure at each sampling point, utilizing standard free Gibbs energy change (Equation (9)) [39]:

(9) $${\Delta G}^{0}=-R\times T\times \ln(K_{e})$$

where ΔG^0^ represents change of the Gibbs free energy (J/mol), R (8.314 J/mol∙K) is the molar gas constant, T is absolute temperature, in K, and K_e_ is apparent separation equilibrium constant expressed by Equation (10):

(10) $$K_{e}=\frac{C_{f}}{C_{p}}$$

### 2.4. Characteristics of Hydrated Cations

**Figure 2:**
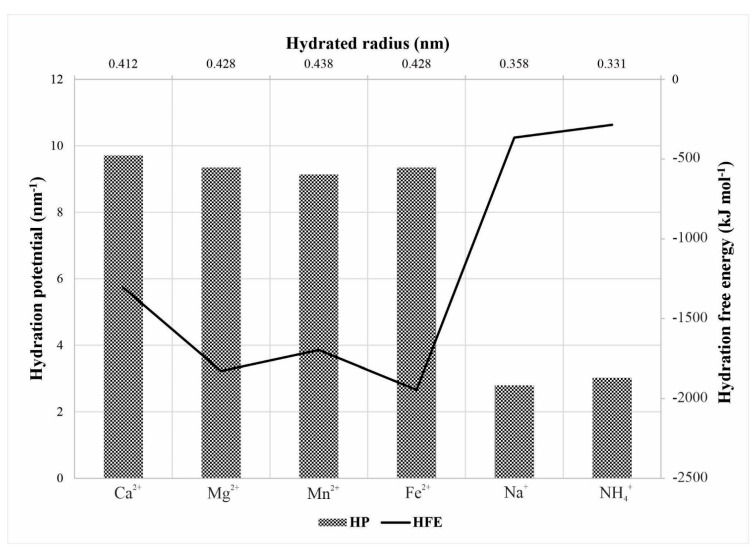
Selected hydration parameters of the investigated cations.

Noncovalent interactions between cations and water molecules are incessant dynamic processes, and cations hydration shells are in continuous rearrangement. The hydration process consists of water molecules transferring from the solution to the hydration shells with a defined substantial ordering around each ion, as found by Ghiu et al. [40]. A cation can strongly or weakly attract water molecules, and hydrated radius (HR) in nm rise depends on the central ion coordination number. The measure of cation attraction intensity toward water molecules is the hydration potential (HP) in nm^−1^ and can be calculated as the quotient of the square of the ionic charge and the ion hydrated radius. In the process of weak bonding among cations and water molecules, energy known as hydration free energy (HFE) in kJ mol^−1^ is released. This exothermic process takes place during formation of the hydration shell. Figure 2 presents the selected characteristics of the investigated hydrated cations in accordance with the literature [10,41,42,43].

The hydrated cations charge density (CD) plays significant role in the size exclusion mechanism [31]. CD is defined as the electric charge per unit volume of the hydrated ion and indicates the charge distribution over the ion volume. Stokes radii (r_S_), Ionic radii (r_i_) and charge densities of the investigated cations are presented in Table 4. CD values in C mm^−3^ were calculated according to the Equation (11):

(11) CD=nic⋅e(43)⋅π⋅ri3

where n_ic_ represents the ion charge, the ionic radii are the Shannon-Prewitt values in millimeters [44], and e is the electron charge (1.60 × 10^−19^ C).

Mobile entity in the water solution, near the membrane surface and through membrane capillaries, occurs as a cation surrounded by a shell of water molecules by weak van der Waals forces rather than just the stripped ion, as concluded by Richards et al. [45]. Consequently, transport through the membrane is controlled by the hydrated size and not by ionic size. In addition, water shells that are weakly bond may devote from the cation and enable its passing through the membrane. Tansel [11] explained that stronger binding exists between high charge density ions and large water clusters compared to those with lower CD ions. Sodium and ammonium, as larger ions, have lower charge densities and manifest less attraction to steric orientation of water molecules in the first solvation shell and represent chaotropes [46]. Investigated divalent cations have smaller ions and higher charge densities with higher hydration potential and can be classified as kosmotropes [46], according to the Collins model prediction [47].

### 2.5. Steric Hindrance Factors

Under the assumption that the cations surrounded by water molecules form spherical hydrated shells, a classic Einstein finding of independent particles moving away from each other with diffusion can be applicable through the Stokes-Einstein equation [48] as a basic of steric exclusion during filtration. The diffusivity of hydrated ions in the pores of nanofiltration dimensions is reduced, and these ions can be sieved during the filtration process. Inherent hindrances to diffusion and convection are referred to as the ion-pore wall and steric limitations. 

The key steric hindrance parameter is relative solute size (q) shown as a quotient of the Stokes cation radius and the effective membrane pore radius in Equation (12).

(12) $$q=\frac{r_{S}}{r_{E}}$$

S_D_ and S_F_ are the steric hindrance factors for diffusion and convection, respectively, and are functions of q, as presented in Equations (13) and (14) [49]. Oren and Biesheuvel defined the steric hindrance factor S_D_ as a partitioning coefficient at the membrane–solution interface [50].

(13) $$S_{D}={\left(1-q\right)}^{2}$$

(14) $$S_{F}=2\cdot {\left(1-q\right)}^{2}-{\left(1-q\right)}^{4}$$

## 3. Results and Discussion

### 3.1. The Cation Rejection and Permeability Behavior of the Membrane

**Figure 3:**
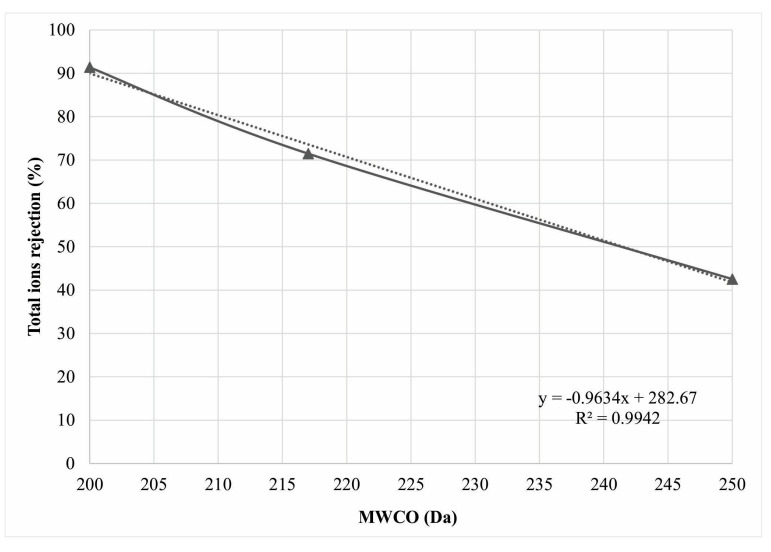
Influence of the different membrane molar cut-off properties to average total ion separation efficiency.

Rejection of total salts, shown as average E_C_ rejection values (Figure 3), in the dependence of nanofiltration pores fineness, i.e., MWCO differentness, shows mutual electrolyte retention levels of the three investigated membrane configurations. Figure 3 shows changes of average investigated cation rejection at different MWCO conditions.

**Figure 4:**
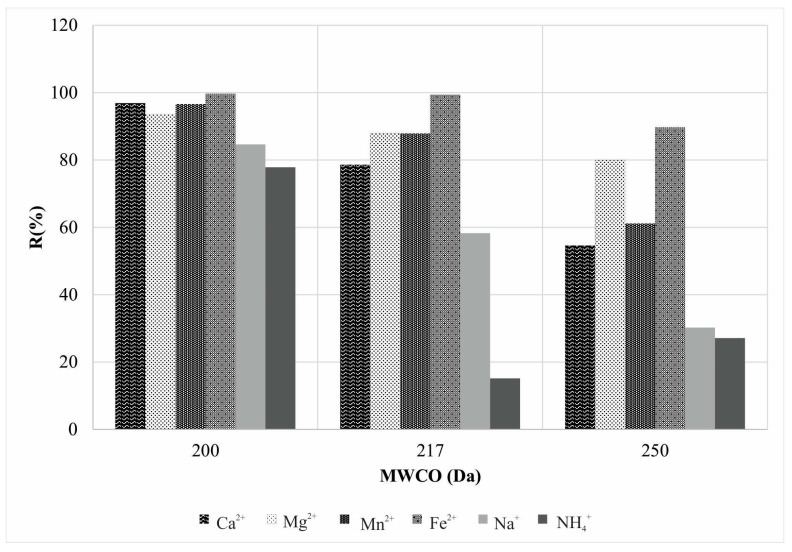
Cation rejection distribution at different filtration fineness.

Correlation of total ions rejection and membrane effective pore radii (Figure 4) showed that a very small range in membrane MWCO values of 50 Da only, with regard to membrane types used, derived permeates of versatile chemical composition. 

In order to define mutual coherence of causal physical units, i.e., membrane pressure and surface area, as well as feed and permeate flow rates, with consequent chemical composition and molar concentration of permeate ionic solutes, the membrane ion rejection affinity dimension -MIRA in bar/% was established as shown in Equation (15):

(15) MIRA=TMP/R

MIRA is normalized TMP with regard to permeate solute moiety and represents the pressure value necessary for a solute rejection change of 1%. Bringing MIRA data and permeate flux utilization contributes to the estimation of the nature of the processes at the membrane surface and in the membrane pores. As the MIRA is lower, the affinity toward to cation removal is higher, from which it follows that more energy (pressure) is needed to permeate the ion.

The plot of MIRA vs. flux efficiency results in a straight line and can be presented as follows in Equation (16):

(16) MIRA=±k×FE±n

**Figure 5:**
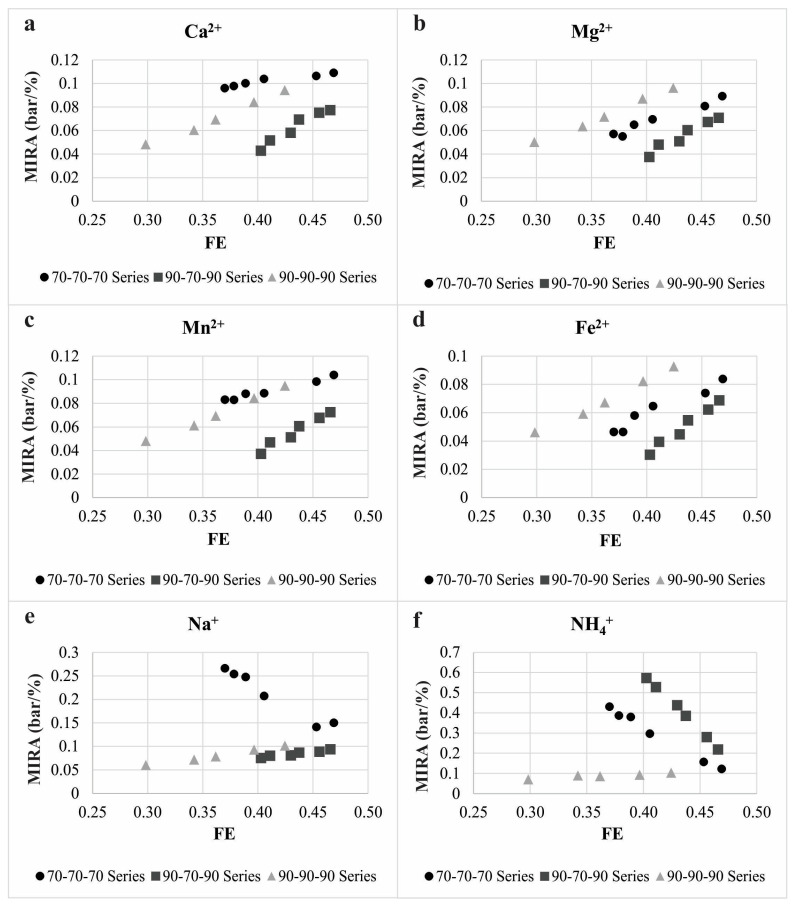
Mutual relation of nanofiltration pressure-driven indicator and ionic molar concentration of calcium (**a**), magnesium (**b**), manganese (**c**), iron (**d**), sodium (**e**) and ammonium ion (**f**) in the dependence of flux utilization.

The plots of MIRA vs. FE for all investigated cations are presented in Figure 5. Slopes (k), intercepts (n), and linear regression measure of strength of the association (r^2^) of all obtained plots, as well as average relative permeabilities, are presented in Table 5.

Extremely low TMP per rejection percent was found for all investigated kosmotropes and depends mostly on the membrane effective pore size, cation charge densities, and hydrated radii. 

Flux and TMP increase had an influence on MIRA enlargement for all investigated cations, but in a different order with regard to membrane effective pore radii. Cations with higher charge density (Mg^2+^_(aq)_ and Fe^2+^_(aq)_) were preferentially rejected in the order of membrane r_E_ as: 0.34 nm < 0.36 nm < 0.33 nm (Figure 5b,d). The MIRA increase for cations with lower CD–Ca^2+^_(aq)_ and Mn^2+^_(aq)_ (Figure 5a,c) was observed in the r_E_ sequence as follows: 0.34 nm < 0.33 nm < 0.36 nm. 

Membrane rejection affinity to monovalent sodium was high for r_E_ of 0.33 nm and 0.34 nm and to ammonium ion in 0.33 nm experiments and depended primarily on charge density. Pore effective radii of 0.36 nm for Na^+^_(aq)_, and 0.34 nm for NH_4_^+^_(aq)_ showed a dominant influence of charge density on MIRA. The extremely high MIRA values for sodium ion at membrane MWCO of 250 Da (Figure 5e) and for ammonium ion at membrane MWCO of 217 Da (Figure 5f) indicated a distinct permeation process of these cations. Na^+^_(aq)_, and NH_4_^+^_(aq)_ ions manifested typical chaotrope behavior through a very low charge density and small hydrated radius. FE increase contributed to monovalent ion MIRA values declination for specific membrane MWCO that was opposite to the divalent cations MIRA trend. This phenomenon probably argues that monovalent ions steric hindrance appears as a consequence of the similarity of hydrated radii to effective membrane pore radii. 

Linear regression measure of strength of the association of all obtained plots was above 0.9 value, which indicates a strong MIRA vs. FE linear relationship. Strong linear relationship was evident due to the way that experimental points were set up. Namely, FE values are influenced by the J_p_ values and MIRA values are proportional to the TMP. Adjustment of concentrate and recirculate flow rates at each experimental point was conducted which contributed to the different feed water pressure thus affecting TMP. Feed water and permeate fluxes were also influenced by the changes at every experimental point. Obtained data in this paper (Figure 5 and Table 5) comply with linear correlation of pressure vs. flux parameters in other experiments using nanofiltration membranes [51,52].

Obtained k and n values depend primarily on membrane system MWCO, cation hydration potential, charge density, and hydration radius. This was indicated by the standard deviation (SD) values which suggested the dispersion of a k (SDk) and n (SDn) set of values (Table 6).

Slope k (Table 5), defined as ion rejection coefficient IRC, represents the rejection capability of a membrane and indicates the prevailed nanofiltration partitioning mechanism near the membrane surface. Positive values of the IRC indicate satisfactory rejection efficiency of the membrane process, and its negative values show very low rejection affinity and high permeability of the membranes for the individual solutes. Negatively signed IRC attributed to negative membrane rejection behavior for some cations (Figure 5e,f). 

Within the 217 Da series, low divalent ions SDk indicated high rejection of Ca^2+^_(aq)_, Mg^2+^_(aq)_, Fe^2+^_(aq)_, and Mn^2+^_(aq)_. IRC value for Na^+^_(aq)_ had a positive sign, but was lower than IRC for divalent ions, showing significantly lower rejection. NH_4_^+^_(aq)_ ions dominantly permeated through the membrane which was demonstrated by a negative IRC value.

The 200 Da MWCO experimental series provided positive IRC values for all investigated ions with very low SDk and high rejection of divalent ions. Na^+^_(aq)_ and NH_4_^+^_(aq)_ ions with higher SDk were majority rejected.

Results obtained in 250 Da series indicated high SDk values for both monovalent and divalent cations. Positive values of IRC for divalent cations indicated dominant rejection of those ions for the difference of monovalent cations with negative signed IRC that were highly permeated. 

Therefore, the obtained results consequently mean that IRC and its SDk values indicate the nature of the ion separation mechanism at the membrane surface. As SDk values are lower, the predominant separation process is a size-based exclusion at the pore opening, i.e., repelling of ions is valuably proportional to membrane system effective pore radii, i.e., MWCO. 

**Figure 6:**
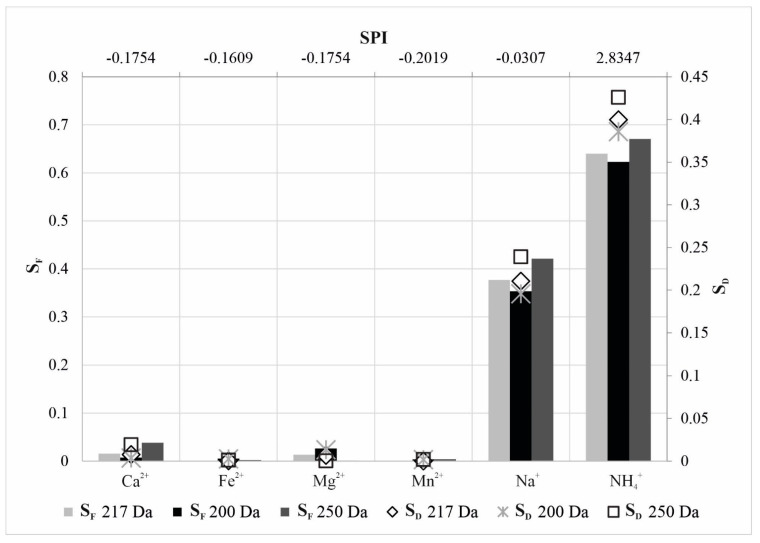
Correlation of steric hindrance factors and solute permeation indicator for investigated cations.

Intercept n value (Table 5) represents a solute permeability indicator (SPI) that shows an ion transport mechanism through the membrane. Negatively signed SPI is associated with less permeable cations and positively signed SPI describes cations of high permeability within defined MWCO membrane configuration. In addition, it is discernible that lower SDn values indicate weaker cation permeation. Obtained results provided the conclusion that the SPI represents a measure of the relative permeability of a particular membrane to a particular solute. The smallest permeation was registered at the membrane system MWCO of 200 Da. The most probable mechanism of the smaller part of the divalent solvents moving through the narrow capillaries of the membrane was a combination of diffusion and electrostatic convection, while the majority of monovalent cations permeated across the membrane pores by diffusion, convection, and electromigration, including significant steric hindrance. The steric hindrance effect was considered only for the sodium and ammonium as ions with the largest Stokes radii (Table 4) [53] and related to their diffusivity in groundwater solution. The dependences of S_F_ and S_D_ vs. SPI are presented in Figure 6.

Enormous enhancement of the steric hindrance factor values for sodium and ammonium ions indicated their high permeation. The SPI values were proportional to S_F_ (r^2^ = 0.9567; r^2^ = 0.9812; r^2^ = 0.7473) and S_D_ values (r^2^ = 0.9752; r^2^ = 0.9925; r^2^ = 0.7943) expressed through a strong linear regression, respectively, for NF3-90, NF3-70 and NF90-70-90 series (refer to Supplementary Data). These high correlations provided good proportion of SPI to q for the series NF3-90, NF3-70, and NF90-70-90 as follows r^2^ = 0.8589; r^2^ = 0.9273; r^2^ = 0.6142, respectively. Therefore, the lowest q, and consequently lowest S_D_ and S_F_ for sodium and ammonium ions, indicate the minimal rejection of these cations. The highest regression coefficients obtained for 250 Da MWCO filtration indicated dominant cation permeation for this pore fineness. High coherence between SPI and steric hindrance factors can predict intensity of different cation permeations and different membrane effective radii. The SPI values enable calculation of the most probable average membrane pores effective radius in a very simple way at the base of the hydraulic and concentration experimental parameters cognition. The dominant size exclusion process is in the case of NF3-90 series where the membrane pore effective radius of 0.33 nm was smaller than hydrated radii of all investigated cations. 

It was discovered that the ammonium ion was most permeable probably due to temporary rearrangement of the water molecules in the hydration shells near the membrane surface. This steric transformation is a consequence of a small, hydrated radius and affinity of narrow hydrophilic membrane pores to permeate ammonium ion. Similar to ammonium ions, sodium-hydrated ions expressed excellent permeability, electrostatically promoted by negatively charged membrane surface.

### 3.2. The Membrane Separation Process Thermodynamics

Setting out from the proposals of nonequilibrium thermodynamics which were originally defined by Onsager [54], the authors of this paper assumed steady state during the cations partitioning at the membrane surface. A state of equilibrium of solute ion molar concentrations between the feed and the permeate stream can occur. Constant TMP, J_f_, J_p,_ C_f_, and temperature during the 30-min lasting time at every investigated point ensured constant permeate solute molar concentration, neglecting concentration polarization. This assumption is very plausible when the experimental conditions are perceived as aged isolated systems with sufficient duration to secure thermodynamic equilibrium [54]. Sodium and ammonium ions favorably permeated (Table 5) because of smaller hydrated radii and smaller hydration energies than investigated divalent cations (Figure 2). This assumption has recently been investigated by Kolev and Freger. Kolev and Freger [55] showed where dynamic simulation of molecular ion uptake by membranes indicated highly localized ions at charged sites and absence of their free movement in the membrane phase. Particularly, divalent ions make a very strong binding to membrane fixed charges, and their uptake, with regard to binding to fixed charges, was extremely low leading to possible saturation [56]. 

**Figure 7:**
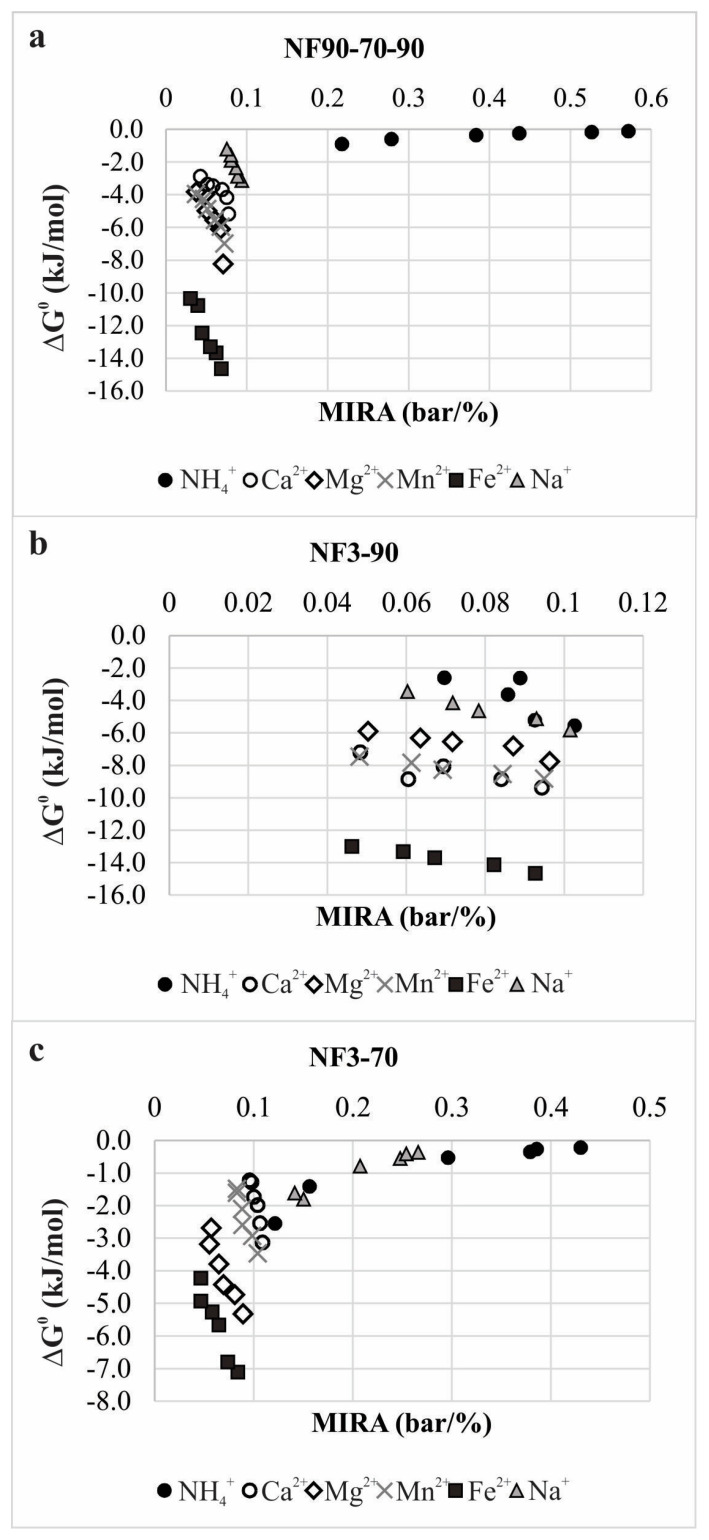
Effects of the membrane ion rejection affinity to changes of the Gibbs free energy at the membrane active layer for NF90-70-90 (**a**), NF3-90 (**b**) and NF3-70 (**c**) experiments.

High hydration energies and larger hydrated radii of calcium, magnesium, manganese, and iron ions contributed to their repel at the membrane surface, as also found by Richards et al. [45]. These monovalent and divalent cations’ different behaviors can also be explained through the adsorption equilibrium process onto a porous membrane charged interface. It was found previously that the adsorption is an ascendant mechanism for separation of cations from the water solution [57]. Energy transferred during phase transformations can be explained by Equation (10). Apparent separation equilibrium constant for all experimental points was higher than one, thus indicating a spontaneous separation process. Effects of the membrane ion rejection affinity to changes of the Gibbs free energy at the membrane active layer are presented in Figure 7.

The average values of the Gibbs free energy changes are found to be proportional to the cations charge density values, in the following order: NH_4_^+^ < Na^+^ < Ca^2+^ < Mn^2+^ < Mg^2+^ < Fe^2+^. Linear correlation of the plots of CD vs. average Gibbs free energy changes showed that regression coefficients r^2^ were 0.7453, 0.8929, and 0.9089 for NF3-90, NF90-70-90, and NF3-70 series, respectively. These highly correlated dependencies indicated that influence of the cations charge density to ΔG^0^ amounts increased with declination of the membrane effective pore radii. The average Gibbs free energy changes were lower than the HFEs for all investigated cations for all applied membrane configurations. With higher ΔG^0^, cation rejection was more exothermic and valuably enlarged. Low ΔG^0^ values near the endothermic zone indicated expressed ion permeation. Besides, when MIRA is lowest, the spontaneity and exothermicity of the cation rejection process is higher. In addition, high permeation of sodium and ammonium ions was indicated by increased MIRA values at very low ΔG^0^ numbers near zero.

## 4. Conclusions

Linking the TMP as a leading nanofiltration process hydraulic parameter and feeding and permeating solute molar concentration established the membrane ion rejection affinity unit-MIRA. Correlativity of MIRA and effective flux contributed to the introduction of important coefficients which explained cation rejection intensity and permeability behavior. Despite the fact that the known experimental law of TMP increase leads to increase of ion repulsion, the quotient of TMP and the efficiency of rejection for individual cations showed upward and downward trends along with flux utilization increase. This phenomenon is determined by the mathematical sign of ion rejection coefficient and is a consequence of the membrane’s pore surface and fineness nature, as well as properties of the hydrated cations. Positive IRC values indicated a pronounced solutes rejection and negative values indicate a very low or negative rejection of individual hydrated ions. The second derived unit-SPI indicated the intensity and nature of ion permeability through the membrane capillaries. From the pronounced correlation of SPI and the steric hindrance factors values, it is possible to predict the most probable effective pore radius of nanofiltration membranes. The dependence of changes in Gibbs free energy and MIRA provided insight into rejection and permeation behavior, distinctive for each investigated cation. It was also found that lower MIRA values indicated higher spontaneity of filtration process, which is strongly coherent with the charge density of hydrated cations and highly dependent on membrane porosity. 

## Figures and Tables

**Table 1:** Monitored physical–chemical characteristics of investigated groundwater.

Parameter	Concentration	Molar Concentration
Temperature	289 K	
pH	7.39	
Ca^2+^_(aq)_	80.08 mg L^−1^	1.998 mmol L^−1^
Mg^2+^_(aq)_	37.11 mg L^−1^	1.526 mmol L^−1^
Mn^2+^_(aq)_	0.332 mg L^−1^	0.006 mmol L^−1^
Fe^2+^_(aq)_	1.79 mg L^−1^	0.032 mmol L^−1^
Na^+^_(aq)_	80.67 mg L^−1^	3.509 mmol L^−1^
NH_4_^+^_(aq)_	3.24 mg L^−1^	0.180 mmol L^−1^

**Table 2:** Main components of nanofiltration pilot plant.

Component	Characteristics	Manufacturer
Microfilter for inlet water pretreatment	Polypropylene filter cartridge of 5 µm with housing	Atlas
Booster pump	Centrifugal multistage pump	Grundfos
	CR1-23; Q = 1.8 m^3^/h; H = 104 m	
Nanofiltration modules	NF membranes	Torey-Korea Inc.
	Φ = 0.102 m; L = 1.02 m	
Instantaneous inlet water, permeate, and concentrate flow meter	Polysulfone rotameter	IBG-Praher
	F1 300–3.000 L/h	
	F2 and F3 200–2.000 L/h	
	F4 100–1.000 L/h	
Water pressure meter	Pressure gauge	Wika
	0–10 bar (M1, M2 and M5)	
	0–20 bar (M3 and M4)	
Solenoid valve	EV220A NC; ¾ inch	Danfoss
Electric control unit	Programmable logic controller	Omron

**Table 3:** Investigated nanofiltration element combinations.

Series	First Stage	Second Stage	MWCO (Da)	r_E_ (nm)
NF3-90	NE90 and NE90	NE90	200	0.33
NF90-70-90	NE90 and NE70	NE90	~217 *	0.34
NF3-70	NE70 and NE70	NE70	250	0.36

* Calculated as average of the MWCO data for one NE70 and two NE90.

**Table 4:** Stokes and Ionic radii and charge densities of the investigated ions.

	Ca^2+^	Mg^2+^	Mn^2+^	Fe^2+^	Na^+^	NH_4_^+^
r_S_ (nm)	0.310	0.347	0.368	0.344	0.184	0.125
r_i_ (nm)	0.100	0.066	0.086	0.075	0.117	0.148
CD (C mm^−3^)	52	120	114	181	24	11

**Table 5:** Average relative permeabilities, slopes, intercepts (n), and linear regression measure of all obtained MIRA vs. FE plots.

Series		Ca^2+^_(aq)_	Mg^2+^_(aq)_	Mn^2+^_(aq)_	Fe^2+^_(aq)_	Na^+^_(aq)_	NH_4_^+^_(aq)_
NF90-70-90	k	0.5483	0.4997	0.5333	0.5807	0.2648	−5.6061
	n	−0.1754	−0.1609	−0.1754	−0.2019	−0.0307	2.8347
	r^2^	0.9519	0.9514	0.9674	0.9759	0.9271	0.9977
	RP (%)	21.4214	12.0934	12.1406	0.6611	41.7400	84.8765
NF3-90	k	0.3737	0.3746	0.3780	0.3759	0.3358	0.2338
	n	−0.0649	−0.0628	−0.0662	−0.0675	−0.0414	0.0027
	r^2^	0.9916	0.9941	0.9938	0.9938	0.991	0.9009
	RP (%)	3.1002	6.4259	3.3866	0.3352	15.3554	22.1605
NF3-70	k	0.1200	0.3213	0.2074	0.3555	−1.2991	−3.1300
	n	0.0529	−0.0626	0.0056	−0.0838	0.7448	1.5808
	r^2^	0.9361	0.9646	0.9733	0.9444	0.9581	0.9910
	RP (%)	45.4451	20.0584	38.8711	10.3073	69.7492	72.8910

**Table 6:** Standard deviation dispersion values of all investigated ions.

MWCO (Da)	217	200	250
**SDk**			
Divalent ions	0.03363	0.00187	0.1079
Monovalent ions	4.15135	0.07212	1.2946
Total ions	2.48941	0.05691	1.40119
**SDn**			
Divalent ions	0.01709	0.00227	0.06282
Monovalent ions	2.02614	0.03118	0.59114
Total ions	1.21954	0.02759	0.66826

## Data Availability

Not applicable.

## Supplementary Materials

The following supporting information can be downloaded at: https://www.mdpi.com/article/10.3390/membranes12010079/s1, Figure S1: Linear correlation of solute permeability indicator vs. steric hindrance factors for diffusion (a) and convection (b).
