# Supplementary material for: Intrinsic Dependence of Groundwater Cation Hydraulic and Concentration Features on Negatively Charged Thin Composite Nanofiltration Membrane Rejection and Permeation Behavior
Source: Membranes (Basel). 2022 Jan 10;12(1):79. doi: 10.3390/membranes12010079 (PMC8781953; doi:10.3390/membranes12010079)
Supplement: Supplementary file 1 [file membranes-12-00079-s001.zip › Supplementary Figure S1.pdf]

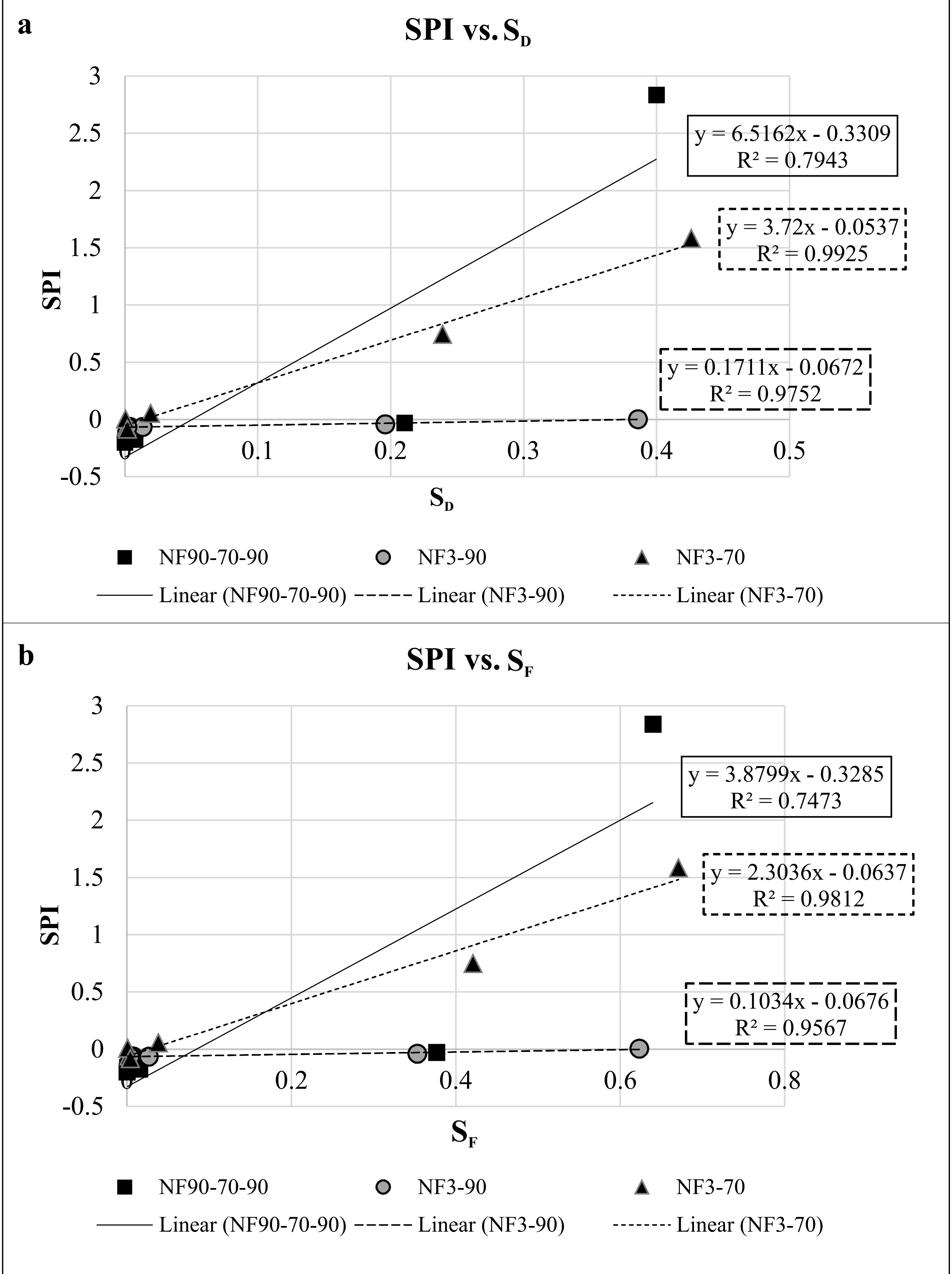


**Figure S1.** Linear correlation of solute permeability indicator vs. steric hindrance factors for diffusion (a) and convection (b)
